# Taxonomic studies on *Begonia* (Begoniaceae) in Myanmar I: three new species and supplementary description of *Begonia rheophytica* from Northern Myanmar

**DOI:** 10.3897/phytokeys.138.38721

**Published:** 2020-01-10

**Authors:** Mya Bhone Maw, Hong-Bo Ding, Bin Yang, Pyae Pyae Win, Yun-Hong Tan

**Affiliations:** 1 Southeast Asia Biodiversity Research Institute, Chinese Academy of Sciences & Center for Integrative Conservation, Xishuangbanna Tropical Botanical Garden, Chinese Academy of Sciences, Menglun, Mengla, Yunnan 666303, China Southeast Asia Biodiversity Research Institute, Chinese Academy of Sciences & Center for Integrative Conservation Yunnan China; 2 Center of Conservation Biology, Core Botanical Gardens, Chinese Academy of Sciences, Menglun, Mengla,Yunnan 666303, China University of Chinese Academy of Sciences Beijing China; 3 University of Chinese Academy of Sciences, Shijingshan District, Beijing 100049, China Core Botanical Gardens, Chinese Academy of Sciences, Menglun Yunnan China; 4 Forest Research Institute, Forest Department, Ministry of Environmental Conservation and Forestry, Yezin, Nay Pyi Taw 05282, Myanmar Forest Research Institute, Forest Department, Ministry of Environmental Conservation and Forestry Nay Pyi Taw Myanmar

**Keywords:** Kachin State, Sect. *Platycentrum*, Putao District, Sect. *Sphenanthera*

## Abstract

Three new species of *Begonia* (*B. chenii*, *B. putaoensis* and *B. crassitepala*) belonging to Begonia section Platycentrum and a supplementary description of *B. rheophytica* with a detailed description of female flowers from Putao, Kachin State, Northern Myanmar, are described and illustrated. All the new species are endemic to Northern Myanmar and can be easily distinguished from other species among the section Platycentrum. A detailed description, photographs, habitat, distribution and a comparison with the most related allied species for all new species are provided.

## Introduction

*Begonia*Linnaeus (1753: 1056) (Begoniaceae) is one of the largest genera of angiosperm in the world, comprising more than 1900 species (Hughes et al. 2015), currently divided into 70 sections (Moonlight et al. 2018). The genus consists of herbs or lianas and is distributed throughout the tropical and subtropical regions of the world (Doorenbos et al. 1998; Moonlight et al. 2018). It has around 959 species and 19 recognized sections in Asia with the bulk occurring in Southeast Asia (Doorenbos et al. 1998; Shui et al. 2002; Ku et al. 2007; Hughes 2008; Moonlight et al. 2018). According to a recent updated checklist of *Begonia* from Myanmar by Hughes et al. (2019), 73 species of *Begonia* have been recorded from Myanmar.

During floristic surveys of northern Myanmar from 2016 to 2018, some interesting *Begonia* specimens were collected. After conducting a detailed examination of the morphological characteristics of the collected material, reviewing the type specimens and taxonomic publications, the authors have confirmed that the specimen of *Begonia* collected from northern Myanmar belong to species new to science, which are described and illustrated below.

Historically, based on the characters of axial placentation, 3 or 4-locular ovary, berry-like and wingless fruit, *Begonia chenii* should belong to Begonia sect. Sphenanthera (Hasskarl 1856: 139) Warburg (1894: 141). However, the recent molecular research result showed that B. sect. Sphenanthera was included in B. sect. Platycentrum (Klotzsch 1855: 243) A. DC. (1859: 134) (Moonlight et al. 2018). As the result, *Begonia chenii*, *B. putaoensis* and *B. crassitepala* belong to B. sect. Platycentrum in the present report.

## Material and methods

Measurements and morphological character assessments of the new species have been examined based on fresh materials and dried specimens. They have been compared with morphologically similar species by affinities inferred using descriptions (Ku et al. 2007, Camfield and Hughes 2018) and type specimens in herbaria (BM, E, K, NYBG, KUN, PE, HITBC and RAF). Protologues and images of type specimens were gathered from JSTOR Global Plants (http:// plants.jstor.org).

## Taxonomic treatments

### Begonia chenii

Taxon classificationPlantaeCucurbitalesBegoniaceae

Y.H.Tan, M.B.Maw & H.B.Ding sp. nov.

CD6E0307-8D39-50DE-B17E-0D65E0A617A0

urn:lsid:ipni.org:names:77204214-1

Figure 1

#### Diagnosis.

*Begonia chenii* Y.H. Tan, M.B. Maw & H.B. Ding is mostly similar to *B. mariachristinae*Wahlsteen (2018: 1) in lanceolate-ovate leaves with silver patches or dots on the upper surface, but significantly differs by stipules slightly pilose (*vs.* glabrous), petiole densely reddish pilose (*vs.* sparsely puberulous), 6 (rarely 4 or 7) tepals of female flower (*vs.* 4 tepals) and red, 3 or 4 locular ovary (*vs.* green, 2 locular).

#### Type.

Myanmar. Kachin State: Putao District, on the way from Putao to Upper Shankhaung, in tropical rain forest, 27°25'36.87"N, 97°16'13.56"E, 512 m, 4 May 2017, *Y.H. Tan*, *B. Yang*, *H.B. Ding*, *X.D. Zeng*, *M.B. Maw & T.S. Tin M1378* (holotype: HITBC!; isotypes: RAF!).

#### Description.

Perennial herb, dioecious or rarely monoecious, lacking rhizome or tuber. **Stem** erect, 40−60 cm tall, reddish brown, densely white pilose, internode 2−11 cm long, branching. **Stipule** persistent, ovate, 1–15 × 3–5 mm, papery, keeled, apex cuspidate (1–4 mm), margin entire, slightly pilose. **Leaf** alternate, petiole 1.5–3 cm long, reddish-brown, densely reddish pilose; **blade** asymmetric, lanceolate-ovate, 8–11 × 2.5–4 cm, apex attenuate, base oblique, margin serrate and with reddish hispid, venation palmate-pinnate, 5−6 pairs of veins; **upper surface** green or dark green with white patches and dots between the veins, bright green shot with metallic blue depending on the angle of the light, especially on young leaves, sparsely reddish hispid, especially along the midrib and lateral veins; **lower surface** deep red or deep red with light green areas both margin linings, scattered reddish hispid and densely along the midrib and lateral veins. **Inflorescence** axillary, flower solitary or in a simple cyme, pendulous; **bract** persistent, ovate to narrow lanceolate, 4–8 × 2–3 mm. **Staminate flower**: pedicel 0.8–1.1 cm, reddish, glabrous or sparsely pilose; tepals 4 (rarely 5 or 6), reddish with white margins, unequal, inner 2 (rarely 3), ovate, 7–1 × 6–1 mm, glabrous, outer 2 (rarely 3), ovate, 7–9 × 7–1 mm, reddish or whitish pilose on the outer surface, margin entire; androecium actinomorphic, stamens numerous, filament free, anther oblong, golden yellow. **Pistillate flower**: pedicel 0.6–0.8 cm, tepals 6 (rarely 4 or 7), unequal, inner 3, elliptic, 8–10 × 3–4 mm, pink to white, glabrous, outer 3, ovate or elliptic, 7–11 × 5–7 mm, reddish or whitish pilose on outer surface; ovary red, slightly or densely pilose on the surface, triangular or rhomboid winged, 3–5 × 2–5 mm, placentation axillary, locules 3 or 4, placentae 2 per locule; styles 3, fused at base, stigma bifid with twisted bands, highly convolute, yellow or golden yellow. **Fruit** berry-like, red, reddish or whitish pilose, triangular, rhomboid (8–15 × 6–9 mm) or suboblate (8–15 mm in diam.), 3 or 4 horned, rarely wingless.

**Figure 1.**
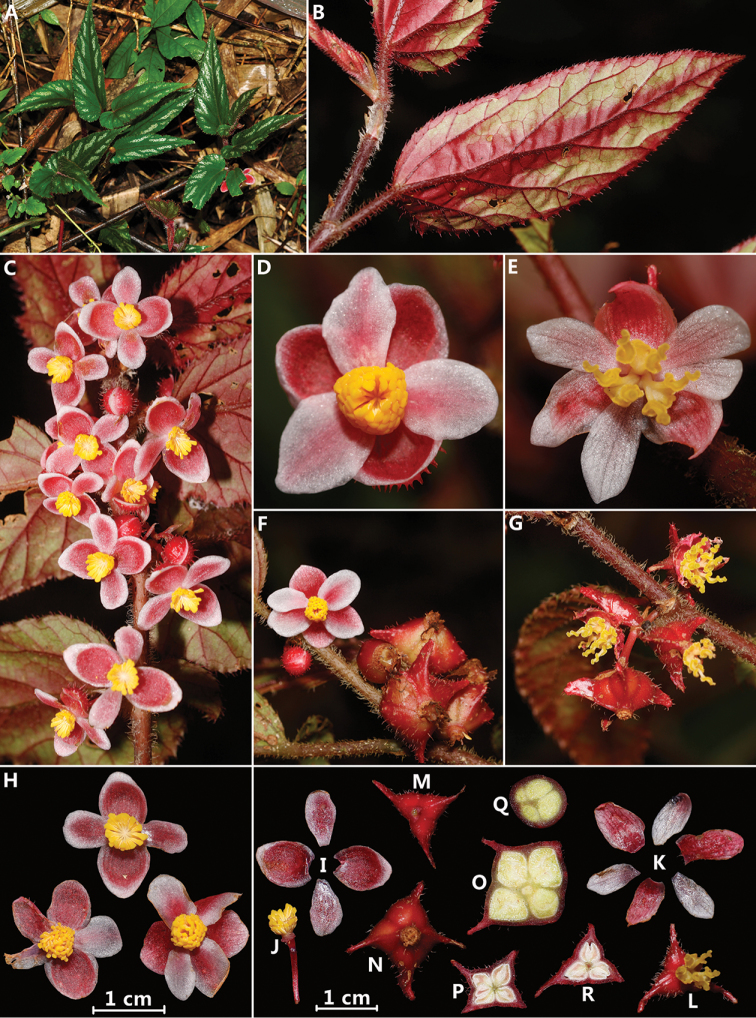
*Begonia chenii* Y.H.Tan, M.B.Maw & H.B.Ding, sp. nov. (photographed by H.B. Ding and Y.H. Tan) **A** habitat **B** leaves (back view) **C** inflorescence **D** staminate flower showing 6 tepals **E** pistillate flower **F** infructescence showing monoecious **G** infructescence showing stigmas **H** staminate flower showing variation of 4, 5, 6 tepals **I** tepals of staminate flower **J** androecium **K** tepals of pistillate flower **L** ovary and stigma **M** ovary (3-winged) **N** ovary (4-winged) **O–P** serial cross section of ovary (locules 4) **Q** serial cross section of ovary (locules 4 and wingless) **R** serial cross section of ovary (locules 3).

#### Phenology.

Flowering from April to May; fruiting from May to June.

#### Distribution.

The species is only known from the type locality, Putao District, Kachin State, Northern Myanmar.

#### Ecology.

The species grows in the moist shaded environment of tropical rain forest, elevation about 512 m.

#### Etymology.

The species epithet “*chenii*” is named after Professor Chen Jin, the director of Southeast Asia Biodiversity Research Institute, Chinese Academy of Sciences, who gave us the opportunity to study the Myanmar flora, which led to the discovery of this new species.

#### Conservation status.

Data Deficient (DD). *Begonia chenii* was collected along the path on the way from Putao to Upper Shankhaung where any signs of major anthropogenic disturbance were noticed in the type locality. However, further explorations are needed for a proper assessment of conservation due to insufficient information on its distribution and population status. Therefore, the species has been preliminarily assigned to Data Deficient (DD) category according to The Guidelines for Using The IUCN Red List Categories and Criteria (IUCN 2017).

#### Additional specimens examined

**(paratypes).** Myanmar. Kachin State: Putao District, Upper Shankhaung, in tropical montane forest, 27°25'36.87"N, 97°16'13.56"E, 512 m, 4 May 2017, *Y.H. Tan*, *B. Yang*, *H.B. Ding*, *X.D. Zeng*, *M.B. Maw & T.S. Tin M1379* (HITBC!); Kachin State: Putao District, Upper Shankhaung, 27°25'35"N, 97°16'14"E, 500 m, 29 April 2016, *Y.H. Tan & S.S. Zhou M201627* (HITBC!); Kachin State: Putao District, Upper Shankhaung, 27°25'34"N, 97°16'13"E, 520 m, 7 May 2017, *S.S. Zhou & X.D. Zeng M2030* (HITBC!; RAF!)

**Affinities.***Begonia chenii* is morphologically similar to *B. mariachristinae*. But it can be easily distinguished in having 3 or 4 locules (*vs.* 2 locules). See Table 1 for detailed comparison of *B. chenii* with morphologically allied species.

**Table 1.** Comparison of key morphological characters of *Begonia chenii* and *B. mariachristinae*.

**Attributes**	***B. chenii***	***B. mariachristinae***
Stem	40–60 cm tall, reddish brown densely white pilose	40–60 cm tall dark red to maroon, hairs
Stipules	persistent, slightly pilose	persistent, glabrous
Petiole	1.5–3 cm long, densely reddish pilose	1–5 cm long, sparsely puberulous
Leaves	lanceolate-ovate, 8–11 × 2.5–4 cm	lanceolate-ovate 6–11.5 × 2.5–4.5 cm
Upper surface	sparsely reddish hispid especially along the midrib and lateral veins	slightly reddish hispid
Lower surface	scattered reddish hispid and densely along the midrib and lateral veins	slightly hairy along midrib
Male flower	tepals 4 (rarely 5 or 6) reddish with white linings	tepals 4 pink or white
Female flower	tepals 6 (rarely 4 or 7) pink to white	tepals 4 pink or white
Ovary	locules 3 or 4 red, slightly or densely pilose	locules 2 green, hispid
Style	3	2 (or 3)

### Begonia putaoensis

Taxon classificationPlantaeCucurbitalesBegoniaceae

Y.H.Tan, M.B.Maw & H.B.Ding sp. nov.

62E88CA6-61CD-5DC4-ABB7-131238C1589B

urn:lsid:ipni.org:names:77204216-1

Figures 2 , 3

#### Diagnosis.

*Begonia putaoensis* Y.H. Tan, M.B. Maw & H.B. Ding is morphologically similar to *B. scintillans*Dunn (1920: 111) in rhizomatous creeping habit and dark green ovate leaves with silver or white area on the upper surface, but it can be easily distinguished by the following characters: sparsely pubescent adaxially leaf (*vs.* densely strigose) and glabrous capsule (*vs.* red villose).

#### Type.

Myanmar. Kachin State: Putao District, on the way from Camp 2 to Camp 3 along Putao to Madwel, on moist rocky slope in tropical rain forest, 27°39'35"N, 97°22'30"E, 505 m, 29 November 2017, *Y.H. Tan*, *B. Yang*, *H.B. Ding*, *X.D. Zeng*, *M.B. Maw & P.K. Linn M2923* (holotype: HITBC!; isotypes: RAF!).

#### Description.

Perennial herb, monoecious, 10–25 cm tall. **Rhizome** creeping with adventitious roots, sometimes branched, 3–30 cm long, 5–12 mm thick, reddish brown, densely pubescent or rusty villous, internode short, 3–8 mm long. **Stipule** triangular, 6–12 × 5–6 mm, apex cuspidate (3–5 mm), margin entire, rusty colored, densely rusty tomentose on both surfaces, persistent; **petiole** scarlet red to crimson, cylindrical, 3–15 cm long, densely rusty tomentose. **Blade** ovate to widely ovate, 6.5–15 cm long, 6–11cm wide, asymmetric, adaxially dark green with gray or light-green areas, slightly pubescent, abaxially light-green, deep red along veins, rarely entirely dark red or red with light-green areas, slightly pubescent, rusty villous on the veins; base cordate, apex acuminate to attenuate, margin sinuate, with sparse hairs; venation palmate, 7–9 veined, adaxial slightly impressed, abaxial distinctly prominent. **Inflorescence** axillary, sub-corymb, erect, branching 2–3 times, 7–19 cm long. Primary peduncle 5.5–14.5 cm long, densely rusty tomentose, dark red, secondary 0.6–1.5 cm long; bract ovate to lanceolate or obovate, 8–13 × 3–7 mm, glabrous, apex acute, margin entire, sometimes with ciliate, 2–10 flowers per inflorescence, male flowers open earlier at the same node. **Staminate flower**: pedicel white or pink, glabrous, 0.9–3.7 cm long, tepals 4, rarely 6, outer 2 (or 3) larger, pink, ovate to suborbicular, 1.3–1.8 × 1.1–2.1 cm, glabrous or abaxially strigose; inner 2 (rarely 3), smaller, white-pinkish, ovate or obovate, 9–17 × 7–11 mm., glabrous; **androecium** 4–6 mm long, 5–7 mm in diameter; **stamens** numerous, filaments ca. 1.7 mm long, anthers yellow, obovate, nearly 1.2 mm long, apex obtuse. **Pistillate flower**: pedicel dark red, glabrous, 2.6–3.3 cm long, tepals 5, rarely 6, outer 3, larger, pink, ovate, 14–16 × 1–13 mm, inner 2 (rarely 3), smaller, white-pinkish, ovate to suborbicular, 12–16 × 8–11 mm; **ovary** glabrous, 2-loculed, placentae axillary, placentae 2 per locule, **styles** 2 or 3, fused at base, stigma bifid with twisted bands, highly convolute, yellow or golden yellow. **Capsule** nodding, ovoid, glabrous, unequally 3-winged; abaxial wing nearly round-rectangular, 13–17 mm broad, lateral wings shorter, 2.5–3.0 mm broad.

**Figure 2.**
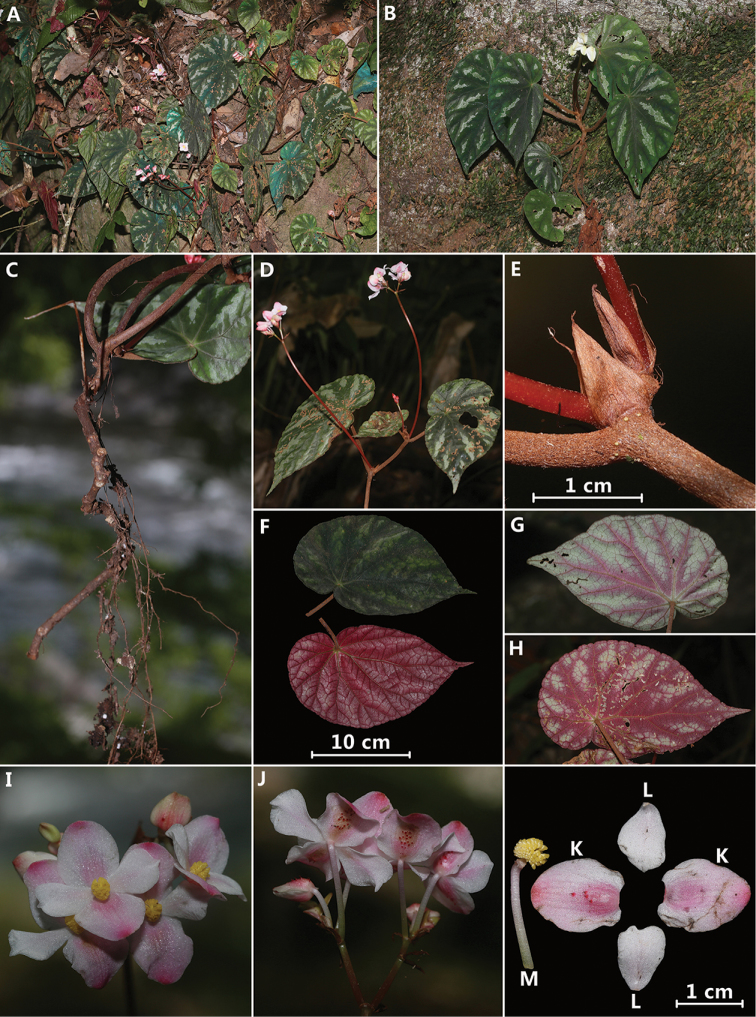
*Begonia putaoensis* Y.H.Tan, M.B.Maw & H.B.Ding, sp. nov. (wild, photographed by H.B. Ding) **A–B** habitat **C** rhizome **D** inflorescence **E** stipule on stem **F–H** single leaf (front and back view) **I** flowers (front view) **J** flowers (back view) **K** outer tepals of male flower (back view) **L** inner tepals of male flower (back view) **M** androecium with pedicel.

**Figure 3.**
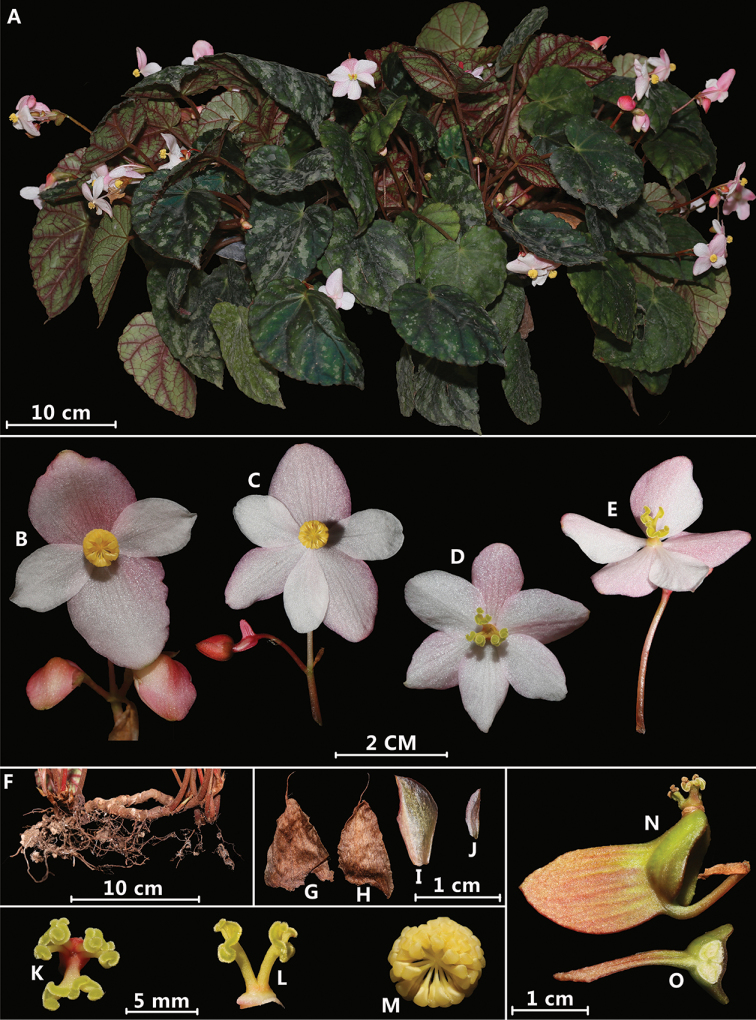
*Begonia putaoensis* Y.H.Tan, M.B.Maw & H.B.Ding, sp. nov. (cultivated plants, photographed by H.B. Ding) **A** habit **B** staminate flower showing 4 tepals **C** staminate flower showing 6 tepals **D** pistillate flower showing 6 tepals and 3 styles **E** pistillate flower showing 5 tepals and 2 styles **F** rhizome **G** stipule (back view) **H** stipule (front view) **I** bract **J** uppermost bract **K** stigma (3, front view) **L** stigma (2, side view) **M** androecium (front view) **N** fruit with unequal wings **O** serial cross section of ovary.

#### Phenology.

Flowering from November to December; fruiting from December to February.

#### Distribution.

The species is known from the single locality in Putao District, Kachin State, Northern Myanmar.

#### Ecology.

The species grows on moist rocky slopes of tropical montane forest, elevation 500–900 m.

#### Etymology.

The species epithet refers to the type locality of the species, Putao District, Kachin State, Northern Myanmar.

#### Conservation status.

Data Deficient (DD). The species might not confront strong human pressures because of the remoteness of its type locality. But we cannot assess the species’ risk of extinction due to lack of data. Therefore, the species is temporarily assigned a status Data Deficient (DD) according to The Guidelines for Using The IUCN Red List Categories and Criteria (IUCN 2017).

#### Affinities.

*Begonia putaoensis* is mostly similar in morphological characters to *B. scintillans* and *B. annulata* K. Koch (1857: 76) under the sect. Platycentrum. But it can be easily distinguished from *B. scintillans* in having shorter internode 0.3–0.8 cm long (*vs.* 3–5 cm long), sparsely pubescent leaf lamina (*vs.* densely hairy) and glabrous capsule (*vs.* red villose). It differs from *B. annulata* through having the following characteristics: rhizomatous creeping (*vs.* rhizomatous erect) and dark green with silver or light green area on upper surface of leaf (*vs.* dark green with silver or white bands). See Table 2 for detailed comparison of *B. putaoensis* with morphologically allied species.

**Table 2.** Comparison of key morphological characters of *Begonia putaoensis*, *B. scintillans* and *B. annulata*.

**Attributes**	***B. putaoensis***	***B. scintillans***	***B. annulata***
Habit	rhizomatous, creeping 15–25 cm tall	rhizomatous, creeping 7–15 cm tall	rhizomatous, erect 15–30 cm tall
Internode	short, 0.3–0.8 cm long	3–5 cm long	0.7–1.5 cm long
Stipule	triangular, 6–12 × 5–6 mm densely rusty tomentose on both surfaces	lanceolate, 6–11 × 4–6 mm villose on outer surface	lanceolate, 4 –13 × 2 – 6 mm tomentose on outer surface
Leaf	ovate to widely ovate 6.5–15 × 6–11cm	ovate-orbicular 4.5–10 × 3.5–7 cm	ovate 9–15 × 5–10 cm
Upper surface	dark green with silver or light green areas sparsely pubescent	dark green with small silver spots, densely strigose	dark green with white/silver bands slightly tomentose or strigose
Lower surface	light green, deep red along veins entire dark-red or red with light green area slightly pubescent, rusty villous on the veins	red, densely red tomentose	red and green, strigose
Staminate flower	tepals 4 (rarely 6), pink or white-pinkish	tepals 4, coral pink	tepals 4, white to pink
Pistillate flower	tepals 5 (rarely 6), glabrous pink or white-pinkish	tepals 4–5, coral pink, pilose on outer surface	tepals 4–5, white to pale pink, puberulous on outer surface to glabrous
Style	2 or 3	3	2
Capsule	ovoid, glabrous, 3-winged longest one round-rectangular 13–17 mm broad	obovoid, red villose, 3-winged longest one rounded oblong 4–6 mm broad	ellipsoid, tomentose, 3-winged longest one rounded oblong 5–9 mm broad

#### Additional specimens examined

**(paratypes).** Myanmar. Kachin State: Putao District, near around Camp 5, along Putao to Madwel, on moist rocky slopes in tropical rain forest, 27°43'59.51"N, 97°22'52.27"E, 873 m, 3 December 2017, *Y.H. Tan*, *B. Yang*, *H.B. Ding*, *X.D. Zeng*, *M.B. Maw & P.K. Linn M3168* (HITBC!; RAF!); MYANMAR. Kachin State: Putao District. Voucher from the cultivated plant in the Xishuangbanna Tropical Botanical Garden, Chinese Academy of Sciences, 12 November 2018, *H.B. Ding XTBG-0050* (HITBC!).

### Begonia crassitepala

Taxon classificationPlantaeCucurbitalesBegoniaceae

Y.H.Tan & M.B.Maw sp. nov.

E6C1FEBD-F706-5D1D-9D70-5DF6C5CF679A

urn:lsid:ipni.org:names:77204217-1

Figure 4

#### Diagnosis.

*Begonia crassitepala* Y.H. Tan & M.B. Maw is morphologically similar to *B. dryadis*Irmscher (1951: 41) in ovate to broadly ovate leaf, but it can be distinguished by its stem and petiole having white prickles (*vs.* puberulous), adaxially leaf having densely pinkish or grey hirsute (*vs.* subglabrous), abaxially outer 2 tepals of pistillate flower and ovary having reddish or whitish succulent strigose and tuberculate (*vs.* puberulous).

#### Type.

Myanmar, Kachin State, Putao District, on the way from Ratbaw to Alanga, 27°17'13.73"N, 97°44'24.28"E, 836 m, 15 June 2018, *Y.H. Tan*, *B. Yang*, *H.B. Ding*, *X.D. Zeng*, *M.B. Maw & H.L. Naing M4495* (holotype: HITBC!; isotypes: RAF!).

#### Description.

Perennial herb, monoecious, rhizomatous. **Stem** erect, 40−60 cm tall, reddish, densely rusty wooly tomentose, and sparsely covered by whitish soft spine-like hairs, internodes 1−2 cm long. **Stipule** lanceolate, 1−1.3 × 0.3−0.5 cm, slightly or densely rusty tomentose, deciduous. **Leaf** petiole 4.5−15 cm, densely rusty tomentose, slightly whitish stiff hairs; **blade** ovate to broadly ovate, base cordate with overlapping lobes, 12−20 × 15−19.5 cm, asymmetric, adaxiall**y** green, densely pinkish or grey hirsute, abaxially green, rusty puberulous, venation palmate-pinnate, densely rusty wooly tomentose along midrib and veins, margin denticulate, reddish hirsute along the margin, apex acuminate. **Inflorescence** nearly terminal, cymose, peduncle 4.4–6.0 cm long, reddish, slightly rusty wooly tomentose and whitish soft spine-like hairs. **Staminate flower**: pedicel 1.8–2.5 cm long, whitish villose, tepals 4, unequal, outer 2, ovate, 2.2−2.7 × 2.6−2.8 cm, whitish or pinkish with pink lining, thick, ca. 2 mm, inner 2, smaller, 1.7 × 2.1 cm, whitish or pinkish (sometimes with pinkish lining), densely whitish villose on the outer surface; stamen numerous, ca. 200; filaments ca. 2 mm long, fused at base; anther oblong to elliptic, 1–2 mm long. **Pistillate flower**: pedicel 2.2–2.5 cm, densely whitish or rusty villose, bracteoles absent; tepals 5, equal, obovate, outer 2, 2.0–2.9 × 1.8–2.0 cm, pure white or sometimes with pink lining, densely pinkish or whitish strigose on outer surface, margin entire, inner 3, similar to outer ones but smaller, 1.8–2.1 × 1.6–1.8 cm, ovary 2-locular, placentation axillary, placentae 2 per locule, densely reddish or whitish succulent strigose and tuberculate, styles 2, fused at base, stigma bifid with twisted bands, highly convolute, yellow or golden yellow. **Fruit** berry-like, elliptic, pale green to pink, 3 wings, unequal, with whitish or reddish succulent strigose and tuberculate (especially on wings).

**Figure 4.**
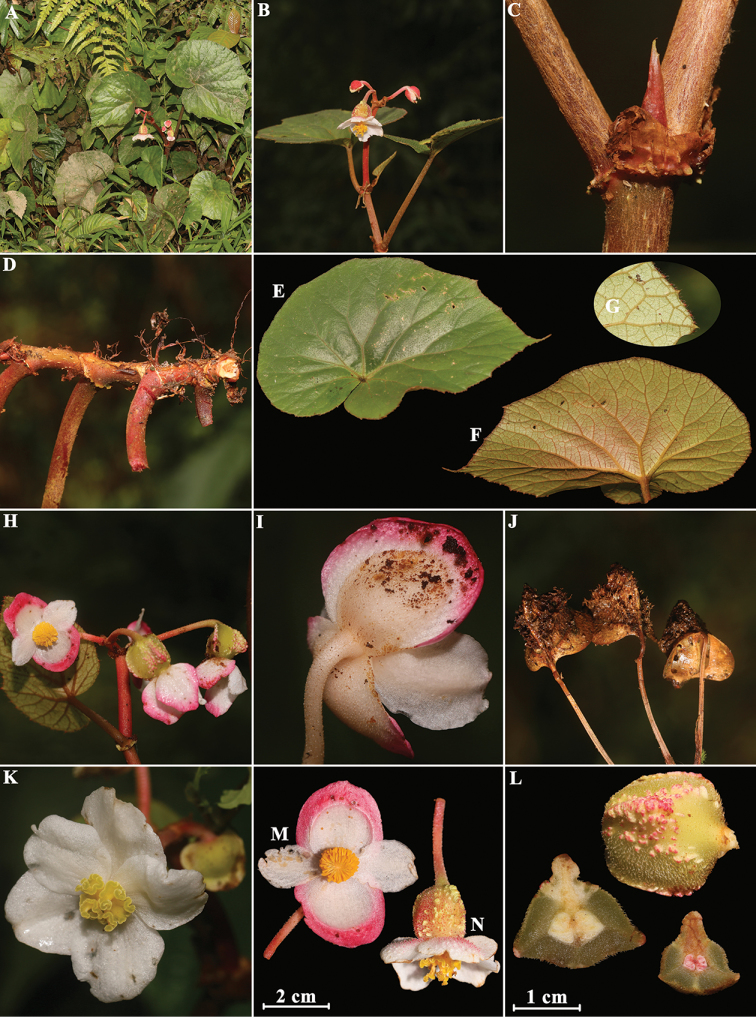
*Begonia crassitepala* Y.H.Tan & M.B.Maw, sp. nov. (photographed by H.B. Ding) **A** habit **B** inflorescence **C** stipule and stem (showing the whitish soft spine-like hairs) **D** rhizome **E** single leaf (front view) **F** single leaf (back view) **G** hirsute leaf margins **H** flowers (front view staminate flower) **I** staminate flower (back view) **J** dried fruits **K** pistillate flower (front view) **M** staminate flower showing 4 tepals **N** pistillate flower (side view) **L** capsule and cross section of ovary.

#### Phenology.

Flowering from June−July; fruiting from July−August.

#### Distribution.

Endemic to the type locality, Putao District, Kachin State, Northern Myanmar.

#### Etymology.

The species epithet refers to its thick tepals.

#### Ecology.

In the tropical montane forest up to about 577 m elevation, on the moist soil slope.

#### Conservation status.

Data Deficient (DD). *Begonia crassitepala* have been collected along the roadside from Langsa to Gawlaw village where no signs of major anthropogenic disturbance were noticed. Further exploration is required to access the current range of the species (IUCN 2017).

#### Additional specimens examined

**(paratypes).** Myanmar. Kachin State: Putao District, along Langsa to Gawlei, tropical montane forest, 27°32'28.94"N, 97°56'36.09"E, 577 m, 2 June 2018, *Myanmar Exped. M3952* (HITBC!; RAF!); Kachin State, Putao District, Gathu to Tongwang Cave, 27°29'53.48"N, 97°58'30.84"E, 664 m, 4 June 2018, *Myanmar Exped. M4008* (HITBC!; RAF!); Kachin State, Putao District, Gathu to Tangsa, 27°28'41.17"N, 97°56'46.40"E, 550 m, 31 May 2018, *Myanmar Exped. M3797* (HITBC!; RAF!); Kachin State, Putao District, Putao Township, Maliraing area, buffer zone of Hkakaborazi National Park, between camp 1 and camp 2, 27°38'03.6"N, 97°22'11.2"E, 552 m, 6 November 2016, *Kate et al. 2253* (NY02653741!); Kachin State, Putao District, Naungmung Township, buffer zone of Hkakaborazi National Park, Hill next to Naungmung village, 27°31'02.2"N, 97°50'46.2"E, 845 m, 12 June 2017, *Kate et al. 2880* (NY02653917!); Between N Dung Ga and Ting Pru Ting Sar, 27 August 1953, *Thar Hla & Chit Ko Ko 4447* (RAF!).

#### Affinities.

*Begonia crassitepala* is the most distinct species in the section Platycentrum thanks to its thicken tepals and succulent strigose and tuberculate ovary. The new species shares similar characteristics with *B. dryadis* in ovate to broadly ovate leaf and 4 tepals of staminate flower. However, it can be easily distinguished by its stem and petiole having rusty tomentose and whitish soft spine-like hairs (*vs.* puberulous), adaxially leaf having densely pinkish or grey hirsute (*vs.* subglabrous), adaxially outer 2 tepals of pistillate flower having densely pinkish or whitish strigose (*vs.* puberulous), ovary having densely reddish or whitish succulent strigose and tuberculate (*vs.* puberulous).

### Begonia rheophytica

Taxon classificationPlantaeCucurbitalesBegoniaceae

M. Hughes, Edinb. J. Bot. 76(2): 2. 2019

507B099A-A942-5722-BCDB-4248CC0A2C77

Figure 5

#### Type.

Myanmar. Hills east of the Mali Hka, 2000−3000 ft, xii 1930, *Kingdon-Ward 9067* (holotype: BM000896328; isotypes: BM000896327, NY02652766).

#### Description.

Herb, rhizomatous, firmly rooted to rock. **Rhizome** 2.5−4.0 cm long and 0.5−1.0 cm in diam., internode 0.2−0.7 cm long. **Stipule** reddish brown, eventually deciduous, narrowly triangular, 0.7−1.0 × 0.3−0.5 cm, keeled, margin entire, glabrous. **Leaf** petiole deep red or deep red to green, turns to brown in mature leaves, sparsely or densely white pilose, 4−17 cm in length, deeply grooved above; **blade** symmetric, narrowly lanceolate, 13.4−18.2(−21) × 2.2−4.0 cm, base attenuate, sometimes unequal, margin red, toothed, teeth tipped by a short ciliate, sometimes undulate, apex elongate; adaxially dark green, glabrous; abaxially pale green, veins densely or sparsely white pubescent; venation pinnate, 5−7 pairs of veins, reddish green in young leaves, red in mature leaves. **Inflorescence** axillary, cymose, peduncle erect, 14−20 cm long, reddish green or pale reddish brown, sparsely hairy; **bract** caducous, broadly ovate−triangular (or ovate−lanceolate), 6−8 × 3−5 mm, purplish red to dark yellow-green, margin entire, hairless. **Staminate flower**: pedicel 1.9−2.1 cm, pale pink (or) pinkish white, sparsely hairy; bracteoles ca. 3 mm, narrowly ovate, dark yellow green, margin entire, hairless, soon falling, **tepals** 4, unequal, inner 2, elliptic, 1.4−1.7 × 0.8−1.0 cm, pure white to pinkish, margin entire, outer tepals 2, broadly ovate, 1.1−1.8 × 1.1−1.5 cm, pure white or pinkish, tip rounded, margin entire; androecium actinomorphic; stamens numerous, filaments fused at base; anther golden yellow, narrowly oblong, apex rounded. **Pistillate flower**: pedicels 2−3 cm, reddish or purplish red, hairless; **bracteole** narrowly ovate 6−8 × 3−5 mm, dark yellow green (sometimes crystal white), soon falling; **tepals** 5−6, unequal, outer 2, equal, broadly ovate, tip rounded, 0.7−1.2 × 0.8−1.2 cm, pure white or white to rosy pink, inner 3 or 4, unequal, 3 larger, 0.8−1.2 × 0.7−1.0 cm, elliptic, pure white or pinkish, tip rounded, 1 smaller, ca. 0.8 × 0.3 cm, pure white, crescent, styles 2, free, stigma bifid with twisted bands, greenish yellow, 4−5 mm long, ovary purplish red, ca. 9−15 mm long, 2−4 mm in diam., wings 3, unequal, placentation axile, locules 2, placentae 2 per locule. **Capsule** nodding, 3-winged, unequal, major wing 8−12 mm long, lateral wings 2−3 mm long.

**Figure 5.**
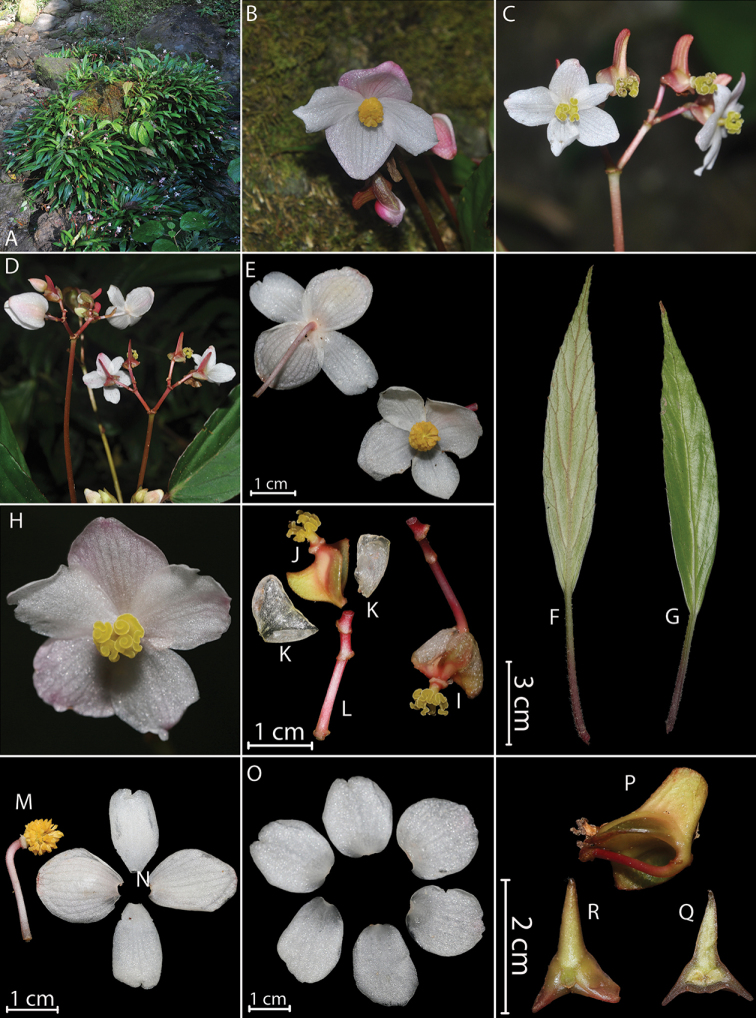
*Begonia rheophytica* M. Hughes (photographed by H.B. Ding and Y.H. Tan) **A** habitat **B** staminate flower (front view) **C** pistillate flowers **D** inflorescences **E** staminate flowers (front and back view) **F** single leaf (back view) **G** single leaf (front view) **H** pistillate flower **I** ovary with gynoecium, pedicel and bracts **J** ovary with gynoecium **K** bracts **L** pedicel **M** androecium with pedicel **N** tepals of staminate flower **O** tepals of pistillate flower **P** capsule **Q–R** cross section of ovary.

#### Distribution.

Only found in Putao District, Kachin State, Northern Myanmar.

#### Additional specimens examined.

Myanmar, Kachin State, Putao District, Camp 1 to Namti (Camp 2), understory herbs in tropical rain forest, 27°24'36.80"N, 97°39'24.38"E, 801 m, 12 December 2018, *Myanmar Exped. M3427* (HITBC!; RAF!); Kachin State, Putao District, near around Camp 1, understory herbs in tropical rain forest, 27°24'18.70"N, 97°36'24.18"E, 850 m, 11 December 2018, *Myanmar Exped. M3334* (HITBC!; RAF!); Kachin State, Putao District, humid rocks near streams or near caves by waterfall of tropical montane forest, 27°24'46.31"N, 93°39'36.28"E, 808 m, 16 December 2017, *Myanmar Exped. M3747* (HITBC!, RAF!).

#### Note.

This species was originally described by Hughes et al. (2019) from the male flowering plant only. Here, we provide a supplementary description of *B. rheophytica* with a detailed monograph of female flowers from Putao, Kachin State, Northern Myanmar.

## Supplementary Material

XML Treatment for Begonia chenii

XML Treatment for Begonia putaoensis

XML Treatment for Begonia crassitepala

XML Treatment for Begonia rheophytica
